# Supplementary material for: A predictive model using the mesoscopic architecture of the living brain to detect Alzheimer’s disease
Source: Commun Med (Lond). 2022 Jun 20;2:70. doi: 10.1038/s43856-022-00133-4 (PMC9209493; doi:10.1038/s43856-022-00133-4)
Supplement: Supplementary file 1 — Supplemental Material [file 43856_2022_133_MOESM1_ESM.pdf]

**Supplementary Material**

## Supplementary Note 1

### *Biological interpretation of ApV*

Our model is structured in two different steps: the first iteration aims to triage people without an AD related pathology from people with a disease who, in the second step, are then finally classified as having a prodromal or late phase of AD. In the radial trees of Figures 2 and 3, regions, features and weights extracted by the algorithm in the two different steps are shown. When discriminating between nADrp and ADrp, LASSO1 extracted 20 features distributed in 14 regions (left lateral ventricle, right hippocampus, left entorhinal, lateral occipital, paracentral, supramarginal, right corpus callosum, fusiform, middle temporal, postcentral, rostral middle frontal, superior frontal, supramarginal and temporal pole). When discriminating between $MCI_{AD}$ and AD, the LASSO2 selected 8 features distributed in the left cerebral white matter, the left and right inferior lateral ventricle, the left cerebellum white matter, the brain stem, the left choroid plexus and the right cerebral cortex. Our results show a predominant dysfunction in the left hemisphere[1]. This confirms the strong left hemispheric lateralization found in the early stages of the disease compared to weak right hemispheric lateralization found in advanced stages[2]. The hippocampus is the first brain region diverging from normal aging models and its atrophy is associated with the enlargements of the lateral ventricles[3]. These two phenomena are confirmed by the results of our model. The Grey Level Co-occurrence matrix (GLCM) correlation feature, filtered with an LHL wavelet filter, in the *left lateral ventricle* expresses the dependency of grey level values to their respective voxels in the GLCM (correlation close to 0: uncorrelated, correlation close to 1: perfect correlation). This might be due to grey levels' distribution in this brain region of AD patients where ventriculomegaly is commonly observed. Brain parenchymal shrinkage causes, in most neurodegenerative disorders, the passive enlargement of the lateral, third and fourth ventricles. Significant ventricular enlargement has been associated with AD[4]. Tangle pathology is thought to spread across the brain in a typical manner, initiating in the transentorhinal cortex, then spreading to the entorhinal and hippocampal areas, and finally extending to the lateral association

cortex[5]. Both right entorhinal cortex and hippocampus are involved in the computation of the $ApV_1$. The *right hippocampus* is described by the Neighbouring Grey Tone Difference Matrix (NGTDM) coarseness feature, which is a measure of the average difference between the central voxel and its neighbourhood and is an indication of the spatial rate of change. A higher value indicates a lower spatial change rate and a locally more uniform texture. Together with high pass wavelet filters applied on one dimension and a low pass one applied on the other two, the extraction of the coarseness in the hippocampus represents an index of heterogeneity. It is the result of cognitive decline, expressed as local neuronal loss of many hippocampal subfields (subiculum, corun ammonis) following AD progression (as also confirmed by the statistically significant decrease of fractional anisotropy). The GLCM variance, extracted in the *left entorhinal cortex,* is also a measure of heterogeneity. This feature infers the presence of intensity level pairs that deviate from the mean and is extracted together with the cluster tendency, a measure of groupings of voxels with similar grey-level values. These features might express the presence, in layer II of the entorhinal cortex, of specific neurons which have proven to show earliest evidence of regional atrophy in the context of age-related memory dysfunction[6]. The volume of the entorhinal cortex is often used to establish the atrophy of neighbouring regions, as the *temporal pole*. In patients with AD, the broad neuronal loss occurring in this area is confirmed here by the extraction of the FOS mean, which describes a global morphological change[7]. While the lateral temporal and parietal cortices exhibit diffuse grey matter loss, some regions of the central and paracentral cortex appear to have several foci of average grey matter loss in territory that is otherwise comparatively spared[8]. This is confirmed by the NGTDM strength feature extracted in the right *paracentral* cortex, a measure of the primitives in an image. This feature results positively weighted in AD brains, describing an image with a slow change in intensity but more large coarse differences in grey level intensities. This might be the result of the multifocal process characterising grey matter loss in that area[8]. The heterogeneity and cortical asymmetry[8] of the left and right *supramarginal gyrus* is inferred by the features extracted in this area: the NGTDM strength, the GLCM correlation, and the FOS median. The NGTDM strength might be a descriptor of the synaptic loss and dysfunction in the parieto-temporal cortex involving the angular gyrus, supramarginal gyrus, inferior parietal lobule causing language function impairment in AD patients[9]. The *superior frontal* gyrus is described by the minimum value of

the fractal dimension and the Grey Level Run Length Matrix (GLRLM) Run Length Non-Uniformity (RLN) feature. Both features are extracted on a low pass filtered image but, unlike the min FD, the RLN is positively weighted. RLN measures the similarity of run lengths throughout the image. With a higher value in AD brains, RLN indicates less homogeneity among run lengths in this area. Together with that, the FD describes a cortical atrophy pattern present in the superior frontal gyrus, which might be the result of the combined effect of AD and aging[10]. Similarly, the min FD extracted in the right *post central gyrus* expresses a morphological change of this area in AD patients where a significantly reduced cortical thickness is present[11]. A similar effect is present in the middle temporal gyrus, whose atrophy has proved to herald the presence of future AD among nondemented individuals[12]. The heterogeneity of this structural change is inferred by the GLCM cluster prominence feature, which is a measure of the skewness and asymmetry of the GLCM. Its positive weight testifies the presence of more asymmetry in AD patients, compared to healthy people. This same feature was also extracted in the *corpus callosum*, together with the FOS mean value. The FOS feature describes a global change occurring in this region and, in more detail, the loss, in AD patients, of intracortical projecting neocortical pyramidal neurons[13]. The asymmetry of this effect, as also confirmed by PET imaging, is inferred by functional decline correlated to the presence of atrophy in specific areas of the corpus callosum[13]. The *fusiform*, responsible for face recognition, is also inferred with the GLCM cluster prominence. The heterogeneity of the GLCM in this area might be related to the presence of clusters of tangles, which lead to abnormal function of the fusiform in MCI and AD patients[14].

Moving to a stricter characterization of AD patients from people with an AD related mild cognitive impairment, a different set of features/regions is involved. *Brainstem* nuclei are interconnected with several cortical structures and regulate several autonomic, cognitive, and behavioural functions. The NGTDM coarseness has been extracted in this region and in the left cerebellum white matter. This might testify local heterogeneity due to significant deformations in the brainstem of AD patients[15]. The FOS minimum was extracted in the *left choroid plexus*. This structure contains epithelial cells which present an active interface between blood and cerebrospinal fluid. The negative weighting of the feature in this area might confirm recent studies which suggest that the choroid plexus-CSF system exhibits

morphological changes and a functional decline with aging, which is believed to be exacerbated in AD patients[16]. Structural change in cortical grey matter has been revealed in several studies[8,17] and often linked to ageing brain and cognitive decline caused by neuronal loss[18]. With LASSO2, the FOS mean absolute deviance and RMS mode of left cerebral white matter and the FOS minimum of the right cerebral cortex were selected. Neuronal density of broaden areas of the brain, such as white matter or cerebral cortex, is compromised in patients with AD[19]. The extraction of first order statistical features in these regions describes global cognitive decline, expressed as local neuronal loss. The FOS features both in the left cerebral white matter and in the right cerebral cortex are evaluated on the low pass filtered image, reflecting global heterogeneity. In fact, white matter abnormalities, and in particular myelin and oligodendrocytes, could be mechanistically important in AD pathology and could be potential treatment targets[20]. The mean of fractal dimension (FD) was extracted in the left inferior lateral ventricle. Fractals are linked to the tortuosity of the surface and the negative weight may indicate a more heterogeneous surface in the AD patients compared to $MCI_{AD}$. The last regions extracted by LASSO2 is the left cerebellum white matter. Traditionally, white matter bundles in the cerebellum are unaffected in AD. Toniolo et al. performed a tractography-based DTI reconstruction of the cerebellum to assess its cognitive role in AD. They found AD patients with lower FA compared to healthy controls in middle cerebellar peduncle, and the left and right superior cerebellar peduncles[21]. Our result confirmed the variation of FA in this area and the algorithm extracted the NGTDM Coarseness of a low pass filtered image, an expression of local heterogeneity.

The method was modified with the integration of cognitive scores (MMSE and LDELTOTAL) and three CSF related biomarkers. With the information given by these scores, the method reached 95% accuracy in the discrimination between nADrd and AD related pathology (90% accuracy in the discrimination between $MCI_{AD}$ and AD patients). As shown in Figures 2 and 3, the highest weights are related to cognitive scores and, without them, features were evenly distributed between left and right brain hemispheres. The hypothesis behind this result is that the prediction is stabilised by structural features. In fact, memory scores, alone, allow a good discrimination between healthy controls and AD, but their performance is worse with inclusion of people affected by earlier forms of the disease

(Supplementary Figure 5). The extracted structural features help to stabilise the prediction and they are distributed in large areas (cerebral cortex and white matter), in the ventricles and in the deep grey matter structures. In the distinction between nADrp and AD related diseases, the algorithm extracted, in addition to regions extracted by the LASSO1 left and right thalamus, right putamen, the corpus callosum. Deep grey matter structures show significantly reduced volume, even in MCI patients[22]. These brain regions are associated with memory, emotional learning, shifting attention, and spatial working memory and they are typically impaired in AD stage[23]. In the *corpus callosum posterior* the method extracted the NGTDM coarseness and the Grey Level Run Length Matrix (GLRLM) Run Length Non-Uniformity (RLN) feature, both evaluated on images blurred in the x and z direction and sharpened in the y direction. RLN measures the similarity of run lengths throughout the image. With a lower value in AD brains (negative weight), RLN indicates more homogeneity among run lengths in the image. In fact, AD patients show thinner corpora callosa than controls, with significant differences occurring within large segments of the splenium, mid-body, and across the genu[24]. The positive coarseness and the negative RLN indicate heterogeneity and, more specifically, lower occurrences of the most predominant pair of neighbouring intensity values. This effect might be due to abnormalities in the architecture and microstructure of WM fibres in AD patients[25] and was also confirmed by the DTI analysis (significant decrease of FA in the corpus callosum posterior). In the characterisation of people with early and late phase of AD, the algorithm appears to be stabilised by features extracted in the fourth ventricle, the CSF, the right ventral diencephalon and the right vessel. The *ventral diencephalon* is not often reported as an area commonly affected by AD. In this region, our study extracted the GLCM coarseness (filtered with an LLL wavelet) and the Grey Level Size Zone Matrix (GLSZM) Small zone low grey level features (filtered with an LLL wavelet). The features are a measure of the inner heterogeneity of this area. In fact, a study on the morphology and distribution of senile and amyloid plaques in AD patients in the striatum and in the diencephalon revealed a different and specific pattern in these areas[26]. The *fourth ventricle,* as previously studied with the lateral ventricle, commonly shows ventriculomegaly in most neurodegenerative disorders following brain parenchymal shrinkage[4]. The mean FOS feature extracted on images blurred in the first and second dimension and sharpened in the third one, infers the average variation of voxel intensity distribution in AD people, compared to

$MCI_{AD}$. The GLCM variance, extracted in the CSF, is a measure of heterogeneity that places higher weights on differing intensity level pairs that deviate more from the mean. The different pairs of intensity levels in the CSF might reflect the presence of different but specific proteins in the CSF and the biochemical and metabolic changes in the central nervous system due to its proximity to the brain. The specific CSF peptides and proteins are β-amyloid 1–42, total tau, and hyperphosphorylated tau. They are linked to the main hallmarks of AD pathology, such as amyloid plaques and neurofibrillary tangles, and can complement clinical examination for the diagnosis of AD[27].

**Supplementary Note 2**

***GWAS interpretation and allele frequencies evaluation***

In terms of GWAS, *TOMM40* SNP *rs2075650* have a body of research associating them with $MCI_{AD}$ and AD[28,29]. Within this study the *rs5756069* SNP was above the significance threshold of $p < 10^{-6}$. In the ApV nADrp vs AD group (Supplementary Figure 6). The *rs5756069* SNP is an intergenic link between *LOC100652763* and *APOL3*. Only in both subcortical and cortical ApVs *TOMM40* was greater than a significance threshold of $p < 10^{-6}$. *TOMM40* encodes a protein that facilitates the movement of proteins into mitochondria. Specifically, *TOMM40* is the channel-forming subunit of a translocase of the mitochondrial outer membrane (*TOM*) that is essential for protein transport into mitochondria. *TOM40* is located adjacent to *APOL*, and the two genes are thought to be correlation with Alzheimer's is due to linkage disequilibrium[30]. Given that after adjusting for *APOE4* allele status, *rs2075650* is no longer significant, this suggests the *TOM40* association signal is driven by the *APOE4* allele and surrounding variants. The *APOL3* is a gene present on chromosome 22 and part of the apolipoprotein L family. The *APOL3* protein is typically located in the cytoplasm and is considered to play a role in the transport of lipids, and/or may facilitate the binding of lipids to organelles. Nine of the currently known apolipoproteins have been detected in the nADrp group. Evidence of substantial mRNA expression has been displayed for eight (*APOC1*, *APOC2*, *APOD*, *APOE*, *CLU*, *APOL2* and *APOL3*)[31]. Whilst the role of *APOL3* in AD has been subject to relatively little research, genes within the *APOL* family comprise of a *BH3* protein domain. This is a characteristic of the *BH3*-only family of pro-apoptosis genes[32,33]. The *BH3* domain is a short peptide that binds to various programmed cell death

proteins, including pro-survival Bcl-2 family members. Previous studies have displayed altered expression of Bcl-2 family proteins in AD vulnerable neurons[34]. Interestingly, rat neurons exposed, in vivo, to β-amyloid have displayed increased expression of *APOL3*[35]. Although the research of *APOL3* within the nADrp is sparse regarding AD, outside of the nADrp the functionality is described. Outside of the nADrp, *APOL3* is the only L family apolipoprotein induced by atherogenic stimuli, such as thrombin, MPO or oxidized LDL; the β-amyloid in AD promotes the formation of atherosclerosis[36]. Knockout of *APOL3* has shown to decrease tubulogenesis, endothelial wound repair, and increases endothelial permeability[37]. Similarly, *APOL3* correlates with inhibition of pro-angiogenic pathways and reduces expression of pro-angiogenic genes. When *APOL3* KO cells were induced with atherogenic stimuli, pro-angiogenic pathways showed part inhibition, Akt phosphorylation increased but not ERK and FAK[37]. Given the connection between *APOL* and Bcl-2, alongside the increase of *APOL3* in mouse neurons β-amyloid, and the role *APOL3* plays in increasing permeability and angiogenesis, this warrants further experimental/perturbation studies of the *APOL3* in control and AD cases.

The GWAS analysis was computed together with the minor allele frequencies (MAF), allele proportions and Hardy-Weinberg Equilibrium Fisher's Exact test p-value and for the SNP *rs2075650* in the ApVs and ApV group for all labels.

With the ApVs classification, the *rs207560* SNP, the minor allele was B and more individuals have a homozygous allele BB in the AD group than the $MCI_{AD}$, and nADrp group (n = 20, 10, 3; MAF = 0.316, 0.278, 0.154). The Hardy-Weinberg Equilibrium p-value was lower in the AD group compared to $MCI_{AD}$ and nADrp (0.856, 1, 1). The proportion of AA alleles was higher in the nADrp group, compared to the $MCI_{AD}$ and AD (145, 74, 91) (Supplementary Table 5A). With the ApV classification, the *rs207560* SNP, the minor allele was B and more individuals have a homozygous allele AA in the $MCI_{AD}$ and nADrp, groups than the AD group (n = 127, 146, 37; MAF = 0.276, 0.146, 0.368). The proportion of BB alleles was lower in the nADrp group, compared to the $MCI_{AD}$, and AD (3, 19, 12) (Supplementary Table 5B).

## Supplementary references


1 Loewenstein, D. A. *et al.* Predominant left hemisphere metabolic dysfunction in dementia. *Archives of Neurology* **46**, 146-152 (1989).

2 Weise, C. M. *et al.* Left lateralized cerebral glucose metabolism declines in amyloid-β positive persons with mild cognitive impairment. *NeuroImage: Clinical* **20**, 286-296 (2018).

3 Coupé, P., Manjón, J. V., Lanuza, E. & Catheline, G. Lifespan Changes of the Human Brain In Alzheimer's Disease. *Scientific Reports* **9**, 3998, doi:10.1038/s41598-019-39809-8 (2019).

4 Apostolova, L. G. *et al.* Hippocampal atrophy and ventricular enlargement in normal aging, mild cognitive impairment (MCI), and Alzheimer Disease. *Alzheimer Dis Assoc Disord* **26**, 17-27, doi:10.1097/WAD.0b013e3182163b62 (2012).

5 Ferreira, D., Wahlund, L. O. & Westman, E. The heterogeneity within Alzheimer's disease. *Aging (Albany NY)* **10**, 3058-3060, doi:10.18632/aging.101638 (2018).

6 Stranahan, A. M. & Mattson, M. P. Selective vulnerability of neurons in layer II of the entorhinal cortex during aging and Alzheimer's disease. *Neural Plast* **2010**, 108190, doi:10.1155/2010/108190 (2010).

7 Galton, C. J. *et al.* Temporal lobe rating scale: application to Alzheimer's disease and frontotemporal dementia. *Journal of Neurology, Neurosurgery & Psychiatry* **70**, 165-173, doi:10.1136/jnnp.70.2.165 (2001).

8 Thompson, P. M. *et al.* Cortical Change in Alzheimer's Disease Detected with a Disease-specific Population-based Brain Atlas. *Cerebral Cortex* **11**, 1-16, doi:10.1093/cercor/11.1.1 (2001).

9 Yun, H. J., Kwak, K. & Lee, J. M. Multimodal Discrimination of Alzheimer's Disease Based on Regional Cortical Atrophy and Hypometabolism. *PloS one* **10**, e0129250, doi:10.1371/journal.pone.0129250 (2015).

10 Bakkour, A., Morris, J. C., Wolk, D. A. & Dickerson, B. C. The effects of aging and Alzheimer's disease on cerebral cortical anatomy: specificity and differential relationships with cognition. *Neuroimage* **76**, 332-344, doi:10.1016/j.neuroimage.2013.02.059 (2013).

11 Yang, H. *et al.* Study of brain morphology change in Alzheimer's disease and amnestic mild cognitive impairment compared with normal controls. *Gen Psychiatr* **32**, e100005, doi:10.1136/gpsych-2018-100005 (2019).

12 Convit, A. *et al.* Atrophy of the medial occipitotemporal, inferior, and middle temporal gyri in non-demented elderly predict decline to Alzheimer's disease☆. *Neurobiology of Aging* **21**, 19-26, doi:https://doi.org/10.1016/S0197-4580(99)00107-4 (2000).

13 Teipel, S. J. *et al.* Progression of Corpus Callosum Atrophy in Alzheimer Disease. *Archives of Neurology* **59**, 243-248, doi:10.1001/archneur.59.2.243 (2002).

14 Cai, S. *et al.* Altered Functional Connectivity of Fusiform Gyrus in Subjects with Amnestic Mild Cognitive Impairment: A Resting-State fMRI Study. *Front Hum Neurosci* **9**, 471, doi:10.3389/fnhum.2015.00471 (2015).

15 Lee, J. H., Ryan, J., Andreescu, C., Aizenstein, H. & Lim, H. K. Brainstem morphological changes in Alzheimer's disease. *Neuroreport* **26**, 411-415, doi:10.1097/WNR.0000000000000362 (2015).

16 Balusu, S., Brkic, M., Libert, C. & Vandenbroucke, R. E. The choroid plexus-cerebrospinal fluid interface in Alzheimer's disease: more than just a barrier. *Neural Regen Res* **11**, 534-537, doi:10.4103/1673-5374.180372 (2016).

17 Fjell, A. M. *et al.* What is normal in normal aging? Effects of aging, amyloid and Alzheimer's disease on the cerebral cortex and the hippocampus. *Progress in neurobiology* **117**, 20-40 (2014).

18 Arendt, T., Brückner, M. K., Morawski, M., Jäger, C. & Gertz, H.-J. Early neurone loss in Alzheimer's disease: cortical or subcortical? *Acta Neuropathologica Communications* **3**, 10, doi:10.1186/s40478-015-0187-1 (2015).

19 Bakkour, A., Morris, J. C., Wolk, D. A. & Dickerson, B. C. The effects of aging and Alzheimer's disease on cerebral cortical anatomy: specificity and differential relationships with cognition. *Neuroimage* **76**, 332-344 (2013).

20 Nasrabady, S. E., Rizvi, B., Goldman, J. E. & Brickman, A. M. White matter changes in Alzheimer's disease: a focus on myelin and oligodendrocytes. *Acta Neuropathologica Communications* **6**, 22, doi:10.1186/s40478-018-0515-3 (2018).

21 Toniolo, S. *et al.* Cerebellar white matter disruption in Alzheimer's Disease patients: a Diffusion Tensor Imaging study. *Journal of Alzheimer's Disease*, 1-10 (2020).

22 de Jong, L. W. *et al.* Strongly reduced volumes of putamen and thalamus in Alzheimer's disease: an MRI study. *Brain* **131**, 3277-3285 (2008).

23 Nie, X. *et al.* Subregional structural alterations in hippocampus and nucleus accumbens correlate with the clinical impairment in patients with Alzheimer's disease clinical spectrum: parallel combining volume and vertex-based approach. *Frontiers in neurology* **8**, 399 (2017).

24 Walterfang, M. *et al.* Shape analysis of the corpus callosum in Alzheimer's disease and frontotemporal lobar degeneration subtypes. *J Alzheimers Dis* **40**, 897-906, doi:10.3233/JAD-131853 (2014).

25 Preti, M. G. *et al.* Assessing Corpus Callosum Changes in Alzheimer's Disease: Comparison between Tract-Based Spatial Statistics and Atlas-Based Tractography. *PloS one* **7**, e35856, doi:10.1371/journal.pone.0035856 (2012).

26 Rudelli, R. D., Ambler, M. W. & Wisniewski, H. M. Morphology and distribution of Alzheimer neuritic (senile) and amyloid plaques in striatum and diencephalon. *Acta Neuropathologica* **64**, 273-281, doi:10.1007/BF00690393 (1984).

27 Dayon, L. *et al.* Alzheimer disease pathology and the cerebrospinal fluid proteome. *Alzheimer's Research & Therapy* **10**, 66, doi:10.1186/s13195-018-0397-4 (2018).

28 Farrer, L. A. *et al.* Effects of age, sex, and ethnicity on the association between apolipoprotein E genotype and Alzheimer disease. A meta-analysis. APOE and Alzheimer Disease Meta Analysis Consortium. *Jama* **278**, 1349-1356 (1997).

29 Osherovich, L. TOMMorrow's AD marker. *Science-Business eXchange* **2**, 1165-1165, doi:10.1038/scibx.2009.1165 (2009).

30 Yu, C. E. *et al.* Comprehensive analysis of APOE and selected proximate markers for late-onset Alzheimer's disease: patterns of linkage disequilibrium and disease/marker association. *Genomics* **89**, 655-665, doi:10.1016/j.ygeno.2007.02.002 (2007).

31 Elliott, D. A., Weickert, C. S. & Garner, B. Apolipoproteins in the brain: implications for neurological and psychiatric disorders. *Clin Lipidol* **51**, 555-573, doi:10.2217/clp.10.37 (2010).

32 Liu, Z., Lu, H., Jiang, Z., Pastuszyn, A. & Hu, C. A. Apolipoprotein l6, a novel proapoptotic Bcl-2 homology 3-only protein, induces mitochondria-mediated apoptosis in cancer cells. *Mol Cancer Res* **3**, 21-31 (2005).

33 Vanhollebeke, B. *et al.* Human Trypanosoma evansi infection linked to a lack of apolipoprotein L-I. *N Engl J Med* **355**, 2752-2756, doi:10.1056/NEJMoa063265 (2006).

34 Kitamura, Y. *et al.* Alteration of proteins regulating apoptosis, Bcl-2, Bcl-x, Bax, Bak, Bad, ICH-1 and CPP32, in Alzheimer's disease. *Brain Res* **780**, 260-269, doi:10.1016/s0006-8993(97)01202-x (1998).

35 Paratore, S. *et al.* Genomic profiling of cortical neurons following exposure to β-amyloid. *Genomics* **88**, 468-479, doi:https://doi.org/10.1016/j.ygeno.2006.06.007 (2006).

36 Gupta, A. & Iadecola, C. Impaired Aβ clearance: a potential link between atherosclerosis and Alzheimer's disease. *Frontiers in Aging Neuroscience* **7**, doi:10.3389/fnagi.2015.00115 (2015).

37 Khalil, A. *et al.* Apoliporotein L3 interferes with endothelial tube formation via regulation of ERK1/2, FAK and Akt signaling pathway. *Atherosclerosis* **279**, 73-87, doi:10.1016/j.atherosclerosis.2018.10.023 (2018).

## Tables and Figures

**Supplementary Table 1:** The recon-all function by FreeSurfer. **a)** List of the 29 pre-processing steps executed by the recon-all function; **b)** A brain mask of 115 brain regions is one of the results of the recon-all function.

**a**

| | |
|---|---|
| Motion Correction | Cut/Fill |
| NU Intensity Correction | Tessellation |
| Talairach | Orig Surface Smoothing |
| Normalization | Inflation |
| Skull Strip | QSphere |
| Automatic Subcortical Segmentation | Automatic Topology Fixer |
| EM (GCA) Registration | Final Surfaces |
| CA Normalize | Cortical Ribbon Mask |
| CA Register | Spherical Inflation |
| Remove neck | Ipsilateral Surface Registration (Spherical Morph) |
| EM Registration, with Skull | Contralateral Surface Registration (Spherical Morph) |
| CA Label | Average Curvature |
| ASeg Stats | Cortical Parcellation |
| Normalization2 | Parcellation Statistics |
| WM Segmentation | |

**b**

| | |
|---|---|
| Background | Left and Right Amygdala |
| Left and Right Cerebral White Matter | CSF |
| Left and Right Cerebral Cortex | Left and Right Accumbens area |
| Left and Right Lateral Ventricle | Left and Right Ventral DC |
| Left and Right Inf Lat Vent | Left Vessel |
| Left and Right Cerebellum White Matter | Left and Right Inferior Parietal |
| Left and Right Cerebellum Cortex | Left and Right Inferior Temporal |

Left and Right Thalamus

Left and Right Caudate

Left and Right Putamen

Left and Right Pallidum

Third Ventricle

Fourth Ventricle

Brain Stem

Left and Right Hippocampus

Left choroid plexus

Fifth Ventricle

WM hypointensities

Non WM hypointensities

Optic chiasm

Corpus Callosum Posterior

Corpus Callosum Mid Posterior

Corpus Callosum Central

Corpus Callosum Mid Anterior

Corpus Callosum Anterior

Left and Right Banks of the Superior Temporal Sulcus

Left and Right Caudal Anterior cingulate

Left and Right Caudal Middle Frontal

Left and Right Corpus Callosum

Left and Right Cuneus

Left and Right Entorhinal

Left and Right Fusiform

Left and Right Isthmus Cingulate

Left and Right Lateral Occipital

Left and Right Lateral Orbitofrontal

Left and Right Lingual

Left and Right Medial Orbitofrontal

Left and Right Middle Temporal

Left and Right Para hippocampal

Left and Right Paracentral

Left and Right Pars Opercularis

Left and Right Pars Orbitalis

Left and Right Pars Triangularis

Left and Right Pericalcarine

Left and Right Postcentral

Left and Right Posterior Cingulate

Left and Right Precentral

Left and Right Precuneus

Left and Right Rostral Anterior Cingulate

Left and Right Rostral Middle Frontal

Left and Right Superior Frontal

Left and Right Superior Parietal

Left and Right Superior Temporal

Left and Right Supramarginal

Left and Right Frontal Pole

Left and Right Temporal Pole

Left and Right Transverse Temporal

Left and Right Insula

**Supplementary Table 2:** Robustness test. The performance of our ApVs when the total and a reduced number of features is considered, was evaluated. The table report the measurements of diagnostic accuracy of $ApV_1$ (**a**) and $ApV_2$ (**b**) obtained when the ApV is computed with the complete set of features extracted by the LASSO (Ftot), the four features with the highest weights (Ftest4) and all the possible permutations with three (Ftest3-p1, Ftest3-p2, Ftest3-p3, Ftest3-p4) and two features (Ftest2-p5, Ftest2-p6, Ftest2-p7, Ftest2-p8, Ftest2-p9, Ftest2-p10).

**a**

| Regions | Features | Ftot | Ftest4 | Ftest3-p1 | Ftest3-p2 | Ftest3-p3 | Ftest3-p4 | Ftest2-p5 | Ftest2-p6 | Ftest2-p7 | Ftest2-p8 | Ftest2-p9 | Ftest2-p10 |
|---|---|---|---|---|---|---|---|---|---|---|---|---|---|
| Left lateral ventricle | GLCM Correl LHL | 0.0812 | | | | | | | | | | | |
| Right hippocampus | NGTDM Coarse HLL | 0.1593 | | | | | | | | | | | |
| Left entorhinal | GLCM sumVar | 0.0244 | | | | | | | | | | | |
| Left entorhinal | GLCM ClTend | 0.0000 | | | | | | | | | | | |
| Left lateral occipital | NGTDM Streng HLL | 0.0384 | | | | | | | | | | | |
| Left paracentral | NGTDM Streng | 0.0834 | | | | | | | | | | | |
| Left supramarginal | FOS Imedian HLL | -0.1052 | | | | | | | | | | | |
| Left supramarginal | NGTDM Streng LLH | 0.0221 | | | | | | | | | | | |
| Right corpus callosum | FOS Imean HLL | -0.2122 | | | | | | | | | | | |
| Right corpus callosum | GLCM ClProm | 0.0855 | | | | | | | | | | | |
| Right fusiform | GLCM ClProm | 0.1032 | | | | | | | | | | | |
| Right middle temporal | FD min HLH | -0.2515 | -0.2515 | -0.2515 | -0.2515 | -0.2515 | | -0.2515 | -0.2515 | -0.2515 | | | |
| Right postcentral | GLCM ClProm | 0.0263 | | | | | | | | | | | |
| Right postcentral | FD min HHH | -0.1238 | | | | | | | | | | | |
| Right rostral middle frontal | GLCM Correl LHL | 0.3764 | 0.3764 | 0.3764 | 0.3764 | | 0.3764 | 0.3764 | | | | 0.3764 | 0.3764 |
| Right superior frontal | GLRLM RLN HHH | 0.0911 | | | | | | | | | | | |
| Right superior frontal | FD min HHH | -0.1782 | | | | | | | | | | | |
| Right supramarginal | GLCM Correl LHL | 0.7572 | 0.7572 | 0.7572 | | 0.7572 | 0.7572 | | 0.7572 | | 0.7572 | | 0.7572 |
| Right supramarginal | GLCM Correl HLL | 0.0249 | | | | | | | | | | | |
| Right temporal pole | FOS Imean LLH | -0.6901 | -0.6901 | | -0.6901 | -0.6901 | -0.6901 | | | -0.6901 | -0.6901 | -0.6901 | |

| | | | | | | | | | | | | |
|---|---|---|---|---|---|---|---|---|---|---|---|---|
| Accuracy | 0.9786 | 0.9615 | 0.9530 | 0.9786 | 0.9530 | 0.9402 | 0.9573 | 0.9145 | 0.9615 | 0.9444 | 0.9615 | 0.9359 |
| AUC | 0.9860 | 0.9803 | 0.9664 | 0.9920 | 0.9805 | 0.9747 | 0.9850 | 0.9616 | 0.9917 | 0.9716 | 0.9861 | 0.9461 |
| Sensitivity | 0.9741 | 0.9483 | 0.9741 | 0.9741 | 0.9569 | 0.9483 | 0.9741 | 0.9052 | 0.9397 | 0.9569 | 0.9483 | 0.9741 |
| Specificity | 0.9831 | 0.9746 | 0.9322 | 0.9831 | 0.9492 | 0.9322 | 0.9407 | 0.9237 | 0.9831 | 0.9322 | 0.9746 | 0.8983 |
| PPV | 0.9826 | 0.9735 | 0.9339 | 0.9826 | 0.9487 | 0.9322 | 0.9417 | 0.9211 | 0.9820 | 0.9328 | 0.9735 | 0.9040 |
| NPV | 0.9748 | 0.9504 | 0.9735 | 0.9748 | 0.9573 | 0.9483 | 0.9737 | 0.9083 | 0.9431 | 0.9565 | 0.9504 | 0.9725 |
| Threshold | 0.6319 | 0.4786 | 0.0737 | 0.0938 | 0.1657 | 0.3118 | -0.0050 | 0.1802 | 0.0969 | 0.1399 | 0.2171 | 0.0000 |

**b**

| Regions | Features | Ftot | Ftest4 | Ftest3-p1 | Ftest3-p2 | Ftest3-p3 | Ftest3-p4 | Ftest2-p5 | Ftest2-p6 | Ftest2-p7 | Ftest2-p8 | Ftest2-p9 | Ftest2-p10 |
|---|---|---|---|---|---|---|---|---|---|---|---|---|---|
| Left Cerebral WM | FOS ImeanAbsDev | 0.0629 | | | | | | | | | | | |
| Left Cerebral WM | FOS RMS LLL | -0.1495 | -0.1495 | -0.1495 | -0.1495 | -0.1495 | | -0.1495 | -0.1495 | -0.1495 | | | |
| Left Inf Lat Vent | FD mean | -0.0133 | | | | | | | | | | | |
| Left Cerebellum WM | NGTDM Coarse LLL | 0.1026 | 0.1026 | 0.1026 | 0.1026 | | 0.1026 | 0.1026 | | | 0.1026 | 0.1026 | |
| Brain Stem | NGTDM Coarse | 0.1028 | 0.1028 | 0.1028 | | 0.1028 | 0.1028 | | 0.1028 | | 0.1028 | | 0.1028 |
| Left choroid plexus | FOS Imin | -0.0262 | | | | | | | | | | | |
| Right Cerebral Cortex | FOS Imin LLL | -0.0185 | | | | | | | | | | | |
| Right Inf Lat Vent | GLRLM RLN HLL | 0.2010 | 0.2010 | | 0.2010 | 0.2010 | 0.2010 | | | 0.2010 | | 0.2010 | 0.2010 |
| Accuracy | | 0.7414 | 0.7069 | 0.7586 | 0.7414 | 0.7414 | 0.7328 | 0.7759 | 0.75 | 0.75 | 0.75 | 0.7328 | 0.7328 |
| AUC | | 0.7999 | 0.7993 | 0.796 | 0.7906 | 0.8026 | 0.7879 | 0.7942 | 0.7966 | 0.7969 | 0.7805 | 0.7766 | 0.7861 |
| Sensitivity | | 0.5 | 0.5741 | 0.4815 | 0.5 | 0.5 | 0.5 | 0.5185 | 0.4815 | 0.5 | 0.4815 | 0.5 | 0.5 |
| Specificity | | 0.9516 | 0.8226 | 1 | 0.9516 | 0.9516 | 0.9355 | 1 | 0.9839 | 0.9677 | 0.9839 | 0.9355 | 0.9355 |
| PPV | | 0.686 | 0.6892 | 0.6889 | 0.686 | 0.686 | 0.6824 | 0.7045 | 0.6854 | 0.6897 | 0.6854 | 0.6824 | 0.6824 |
| NPV | | 0.9 | 0.7381 | 1 | 0.9 | 0.9 | 0.871 | 1 | 0.963 | 0.931 | 0.963 | 0.871 | 0.871 |
| Threshold | | 0.3017 | 0.0215 | 0.2264 | 0.2347 | 0.2618 | 0.2534 | 0.0648 | 0.0914 | 0.4002 | 0.1238 | 0.2492 | 0.2725 |

**Supplementary Table 3**: Summary statistics for alleles on SNP rs207560. The allele proportions and Hardy-Weinberg Equilibrium Fisher's Exact test p-value for the SNP rs2075650 in the ApVs (**a**) and ApV (**b**) group for all labels are reported.

**a**

| | AA | AB | BB | MAF | minor allele | Hardy-Weinberg Equilibrium p-value |
|---|---|---|---|---|---|---|
| $MCI_{AD}$ | 74 | 57 | 10 | 0.2780 | B | 1 |
| nADrp | 145 | 55 | 4 | 0.1540 | B | 0.793 |
| AD | 91 | 82 | 20 | 0.3160 | B | 0.868 |

**b**

| | AA | AB | BB | MAF | minor allele | Hardy-Weinberg Equilibrium p-value |
|---|---|---|---|---|---|---|
| $MCI_{AD}$ | 127 | 95 | 19 | 0.2760 | B | 0.8720 |
| nADrp | 146 | 53 | 3 | 0.1460 | B | 0.5830 |
| AD | 37 | 46 | 12 | 0.3680 | B | 0.8260 |

**Supplementary Table 4:** Model comparison. The performance of our proposed method (in italic) was compared to the models most commonly employed in the literature. The measurements of accuracy are summarised in the table and refer to training and testing of a multivariate analysis perfomed using Random Forest (ensemble bagged trees), Naïve Bayes, K-nearest neighbours (KNN) and Support Vector Machine (SVM).

| | nADrp vs ADrp | | $MCI_{AD}$ vs AD | |
|---|---|---|---|---|
| | train | test | train | test |
| Ensemble Bagged Trees | 87.5 | 31.1 | 66.9 | 11.2 |
| Naïve Bayes | 76.9 | 62.0 | 62.1 | 11.0 |
| KNN | 85.0 | 74.1 | 60.3 | 11.1 |
| SVM | 87.9 | 30.9 | 66.5 | 10.0 |
| *Our method* | *98.3* | *97.8* | *78.7* | *78.6* |

**Supplementary Table 5:** Measurements of diagnostic accuracy of the fluid biomarkers. CSF based biomarkers were collected by the ADNI database and considered as additional feature to test the performance of our classifier. The table reports the measurements of diagnostic accuracy obtained with the application of the established cut-off values (Shawn et al.).

| | Aβ | tau | ptau |
|---|---|---|---|
| Threshold (pg/ml) | 192 | 93 | 23 |
| TP | 108 | 88 | 71 |
| TN | 3 | 4 | 75 |
| FN | 1 | 2 | 19 |
| FP | 87 | 105 | 34 |
| Sensitivity | 0.0091 | 0.9777 | 0.7888 |
| Specificity | 0.9666 | 0.0366 | 0.6880 |
| PPV | 0.2500 | 0.4559 | 0.6761 |
| NPV | 0.4461 | 0.6666 | 0.7972 |
| Accuracy | 0.4422 | 0.4623 | 0.7336 |

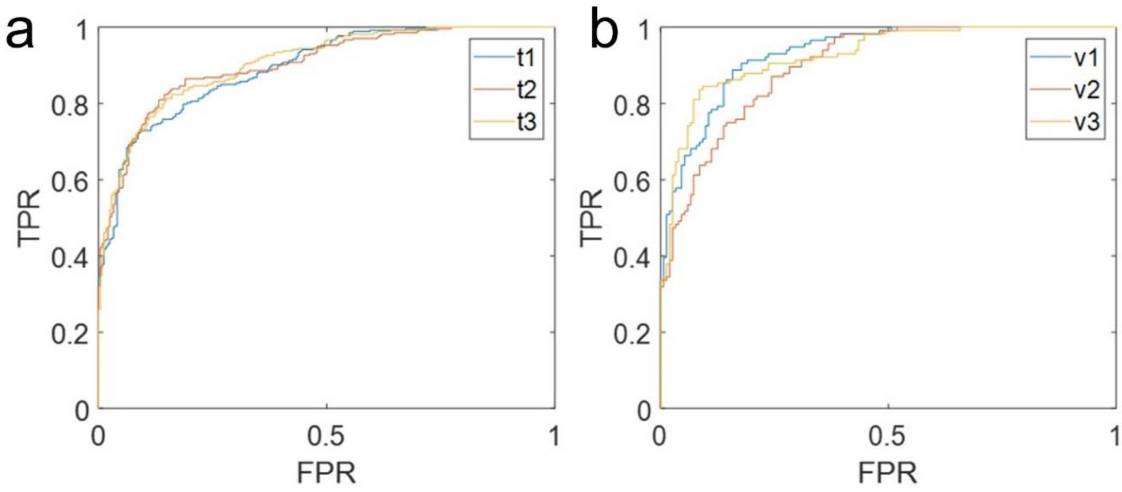


**Supplementary Figure 1**: Effect of splitting training and testing data to include different groups of patients on the model. The model was trained on 1.5T T1w MRI scans obtained from ADNI. With a stratified randomisation, 70% of data was used for training and 30% for testing. The robustness of the randomisation was assessed with the generation of three different sets of train/test datasets. The ROC curves show performance of the datasets used for training (first, second and third sets as t1, t2 and t3, respectively - **a**) and testing (first, second and third sets as v1, v2 and v3, respectively - **b**) the model.

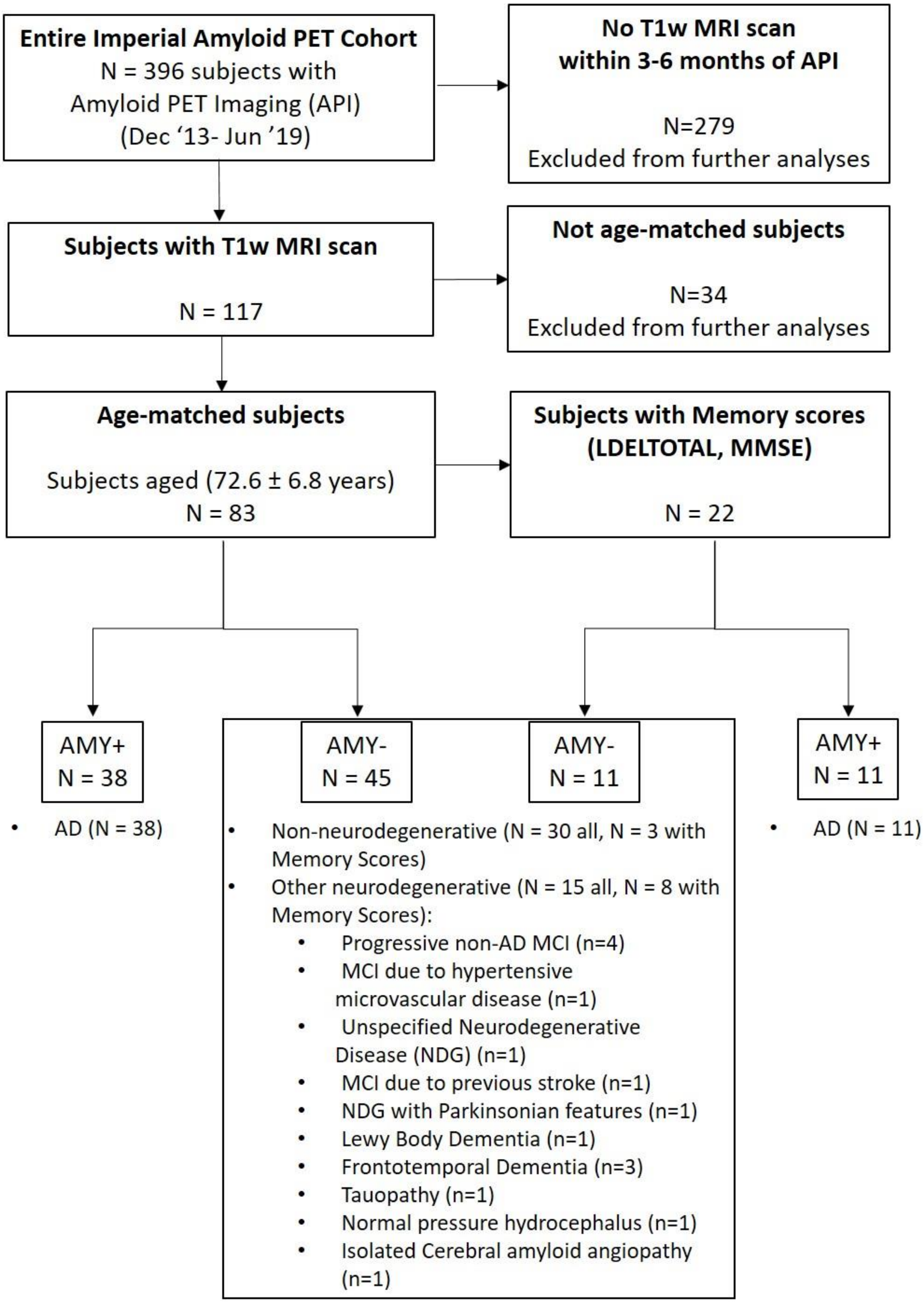


**Supplementary Figure 2:** IMC cohort. The IMC cohort includes 396 patients who underwent, as part of their diagnostic workup, a clinical Amyloid PET scan between December 2013 and June 2019 at the hospital memory centre. The study cohort initially included 117 people for whom a T1w MRI scan

obtained between 3-6 months after Amyloid PET Imaging was available. After the exclusion of age-matching, the final sample available for the analysis consisted of 83 patients, 22 of them with neuropsychological assessment which entailed the administration of the Logical Memory Test (LDELTOTAL) and Mini Mental State Examination (MMSE). All images were visually read as 'amyloid-positive' (AMY+) or 'amyloid-negative' (AMY) by an experienced nuclear medicine radiologist using greyscale images. All AMY+ patients received a clinical diagnosis of AD, while AMY- patients were either diagnosed with another neurodegenerative disease or with a non-neurodegenerative condition (e.g., depression).

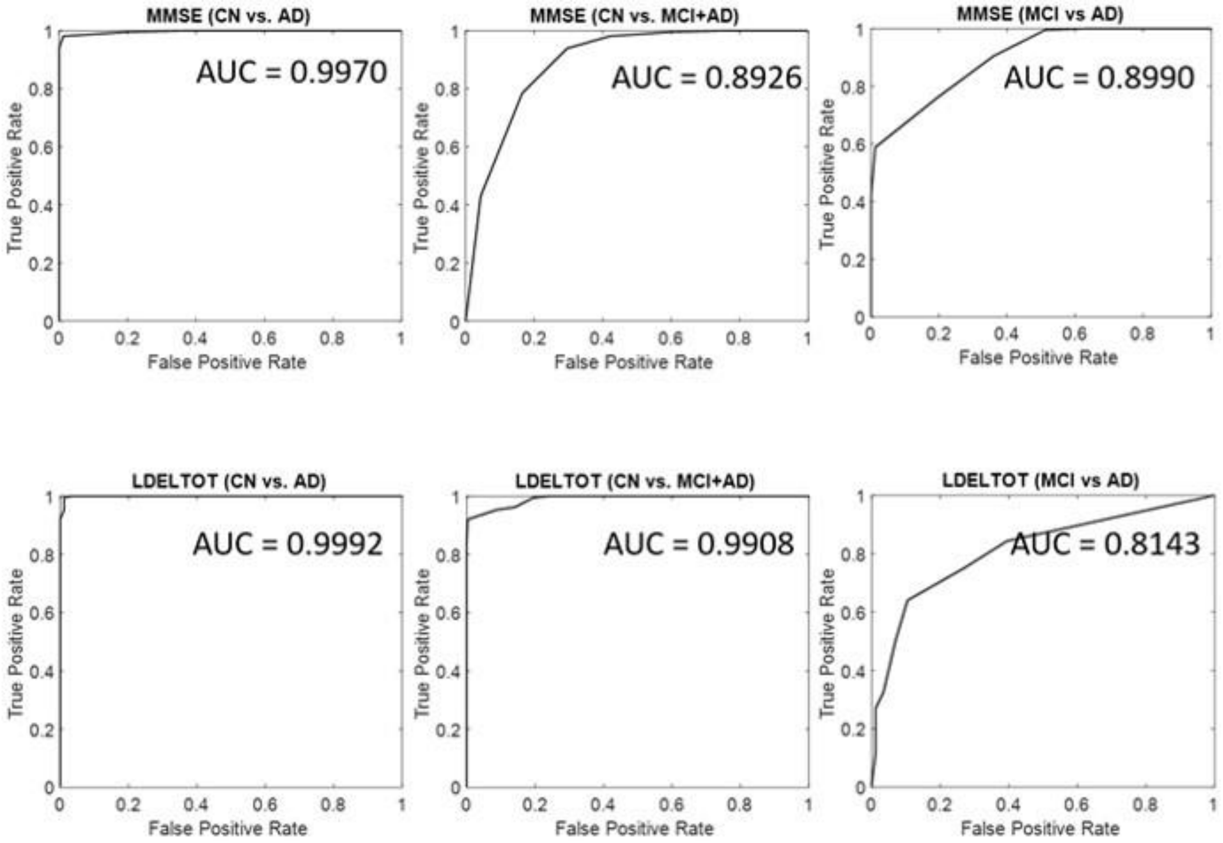


**Supplementary Figure 3:** Cognitive scores**.** ROC analyses of cognitive scores in healthy controls and different stages of AD.

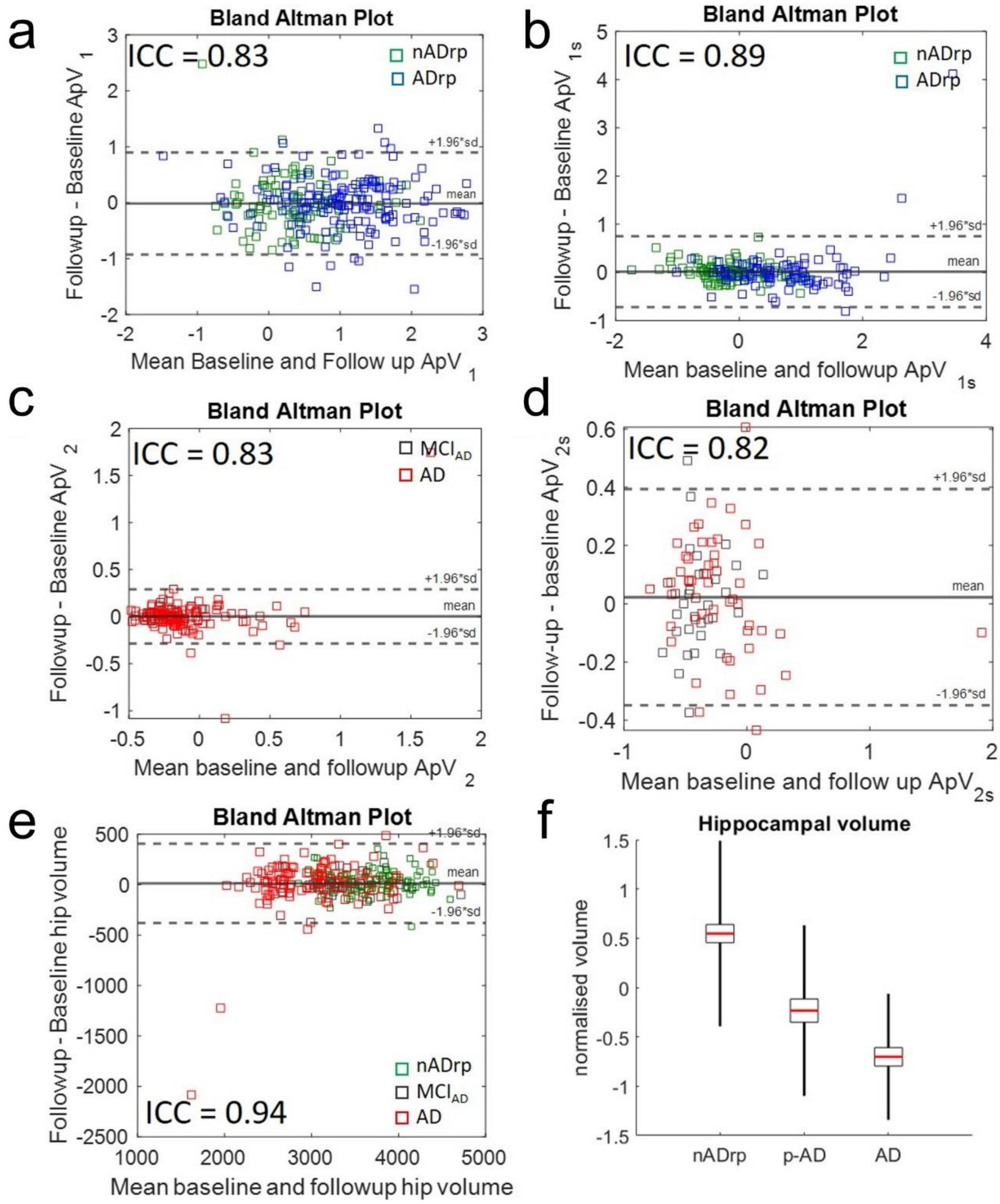


**Supplementary Figure 4**: Test-Retest repeatability and hippocampal atrophy in the ADNI dataset. The ApV methods was compared to a gold standard measure (the volume of the hippocampus) and tested on a second T1w MRI scan obtained on the same day of the baseline scan. The Bland Altman plots report the distribution of $ApV_1$ (**a**), $ApV_{1s}$ (**b**), $ApV_2$ (**c**) and $ApV_{2s}$ (**d**) over the mean (±1.96*standard deviation) and the interclass correlation coefficient. **f**) A boxplot of the distribution of the volumes of hippocampus in the main groups.

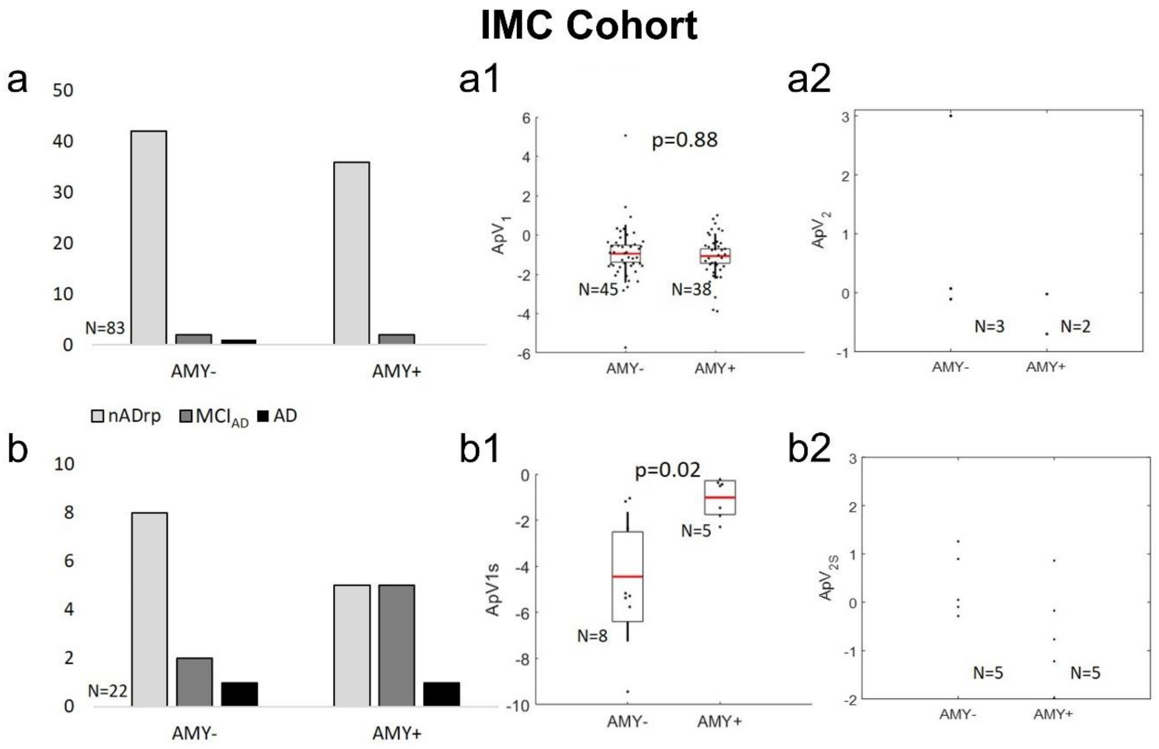


**Supplementary Figure 5**: Validation on the IMC cohort. Our model was tested on a clinical cohort of 83 patients who underwent a clinical Amyloid PET scan and showed negative or positive tracer enhancement (AMY- and AMY+, respectively). AD patients resulted with a positive Amyloid scan, while a negative PET scan resulted from patients with a different neurodegenerative disorder or with no neurodegeneration. The model was tested using the T1w MRI scan acquired with a 1.5T scanner (**a**) and, for a subgroup of patients, with the integration of the LDELTOTAL and MMSE cognitive scores (**b**).

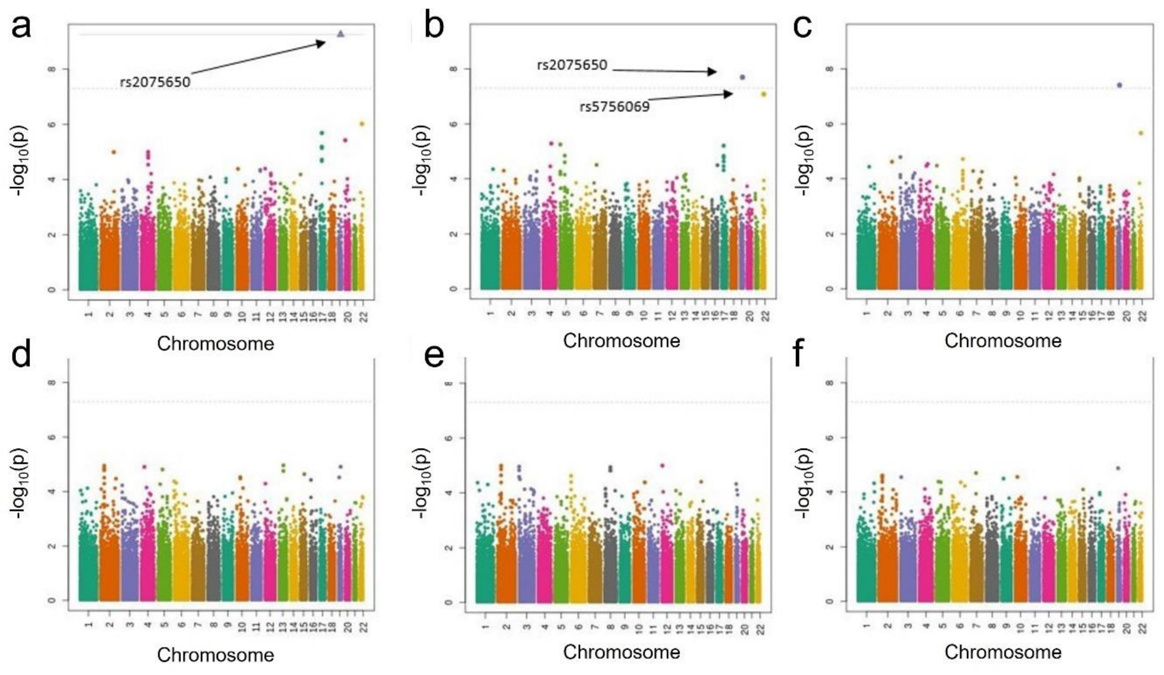


**Supplementary Figure 6**: Manhattan plots of genome-wide association study (GWAS) adjusted for Age and BMI. In panels **a-c** Manhattan plots of the cognitively normal vs Alzheimer's disease derived from the original labels, ApV, and ApVs respectively. In panels **d-f** the Manhattan plot of the cognitively normal vs mild cognitive impairment from the original labels, ApV and ApVs, respectively. The horizontal line displays the cut-off for significant level ($p < 10^{-7}$).

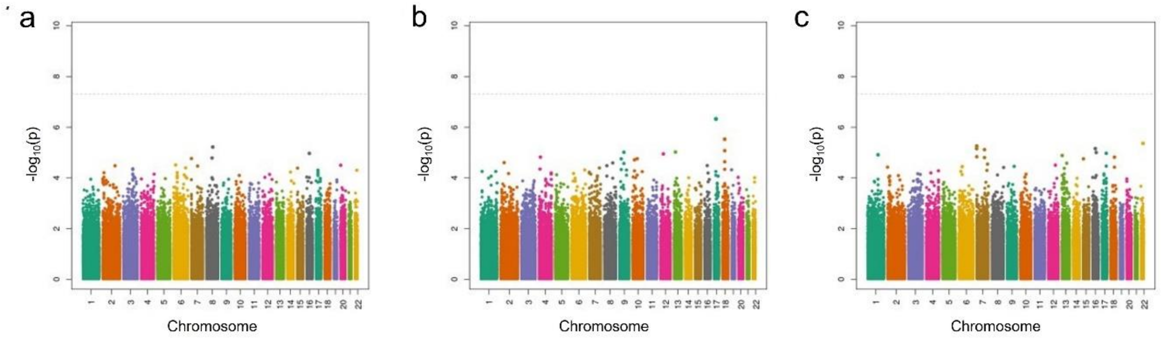


**Supplementary Figure 7**: Manhattan plots of genome-wide association study (GWAS) adjusted for Age, BMI and APOE4 allele status. In panels, **a-c** Manhattan plots of the cognitively normal vs Alzheimer's disease derived from the original labels, ApV and ApVs, respectively.

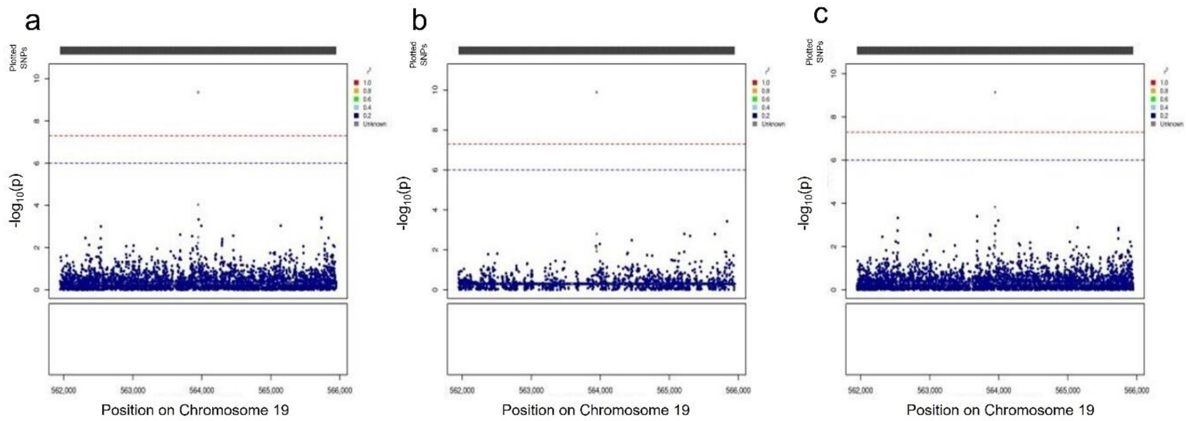


**Supplementary Figure 8**: LocusZoom plots of genome-wide association study (GWAS) adjusted for Age and BMI. In panels A-C: LocusZoom plots of the cognitively normal vs Alzheimer's disease derived from the original labels, ApV, and ApVs, respectively.
