# Supplementary material for: A predictive model using the mesoscopic architecture of the living brain to detect Alzheimer’s disease
Source: Commun Med (Lond). 2022 Jun 20;2:70. doi: 10.1038/s43856-022-00133-4 (PMC9209493; doi:10.1038/s43856-022-00133-4)
Supplement: Supplementary file 4 — Description of Additional Supplementary Files [file 43856_2022_133_MOESM4_ESM.pdf]

Description of Additional Supplementary Files

File Name: Supplementary Data 1

Description: Verifying impact of the algorithm through a literature review. From a Web of Science search, the highly cited (>30 citations) original research articles published in the last 5 years were included in this review. The following key words were used: ("Alzheimer magnetic resonance imaging machine learning" AND "Alzheimer t2 mri features" AND "Alzheimer diffusion mri features" AND "Alzheimer perfusion mri features" AND "Alzheimer blood csf features" AND "Alzheimer blood csf classification" AND "Alzheimer PET classification"). The table also includes the reference to the TADPOLE and DREAM challenges, where the performance of different algorithms at predicting the future evolution of individuals at risk of Alzheimer's is compared. Measures of diagnostic accuracy were reported, when present, and averaged in case of multiple tests. Abbreviations: AC, anterior commissure; AD, Alzheimer's disease; ADNI, Alzheimer's Disease Neuroimaging Initiative; AIBL, Australian Imaging, Biomarker & Lifestyle Flagship Study of Aging; APOE, apolipoprotein E; ASL, arterial spin labelling; AUC, area under the curve; CBF, cerebral blood flow; CBS, corticobasal syndrome; CNN, convolutional neural network; CoT, Contourlet Transform; CuT, Curvelet Transform; CWT, Complex Wavelet Transform; DLB, dementia with Lewy Bodies; DTI, diffusion tensor imaging; DTCWT, Dual Tree Complex Wavelet Transform; DWT, Discrete Wavelet Transform; EMCI, early mild cognitive impairment; EWT, Empirical Wavelet Transform; FA, fractional anisotropy; FBP, Fluorbetapur; FLUTE, Flutemetamol; FTD, frontotemporal dementia; FTP, flortaucipir; HC, healthy controls; HHP, harmonized hippocampal protocol; ICA, independent component analysis; LASSO, least absolute shrinkage and selection operator; LDA, linear discriminant analysis; LMCI, late mild cognitive impairment; lvPPA, logopoeic variant primary progressive aphasia; MAD, median absolute deviation; MCI, mild cognitive impairment; MCS, mild cognitive symptom; MD, mean diffusivity; MMSE, mini mental state examination; MRI, magnetic resonance imaging; NCGG, National Center for Geriatrics and Gerontology; ND, neurodegenerative dementia; NFL, neuronal injury marker neurofilament light; OASIS, Open Access Series of Imaging Studies; RF, random forest; PC, posterior commissure; PCA, principal component analysis; PIB, 11C-labelled Pittsburgh compound-B; pMCI, progressive MCI; pTau, phosphorylated tau; sMCI, stable MCI; rpAD, rapidly progressive AD; rpND, rapidly progressive ND; PCA, posterior cortical atrophy; SCD, subjective cognitive decline; SMC, significant memory concern; ST, Shearlet Transform; SUVR, standardised uptake value ratio; SVM, support vector machine; SWE, stationary wavelet entropy; tTau, total tau.

File Name: Supplementary Data 2

Description: Radiomic analysis. Radiomic features assessed in this study and their corresponding class.
