# Supplementary material for: A predictive model using the mesoscopic architecture of the living brain to detect Alzheimer’s disease
Source: Commun Med (Lond). 2022 Jun 20;2:70. doi: 10.1038/s43856-022-00133-4 (PMC9209493; doi:10.1038/s43856-022-00133-4)
Supplement: Supplementary file 6 — Reporting Summary [file 43856_2022_133_MOESM6_ESM.pdf]

Corresponding author(s): Eric O Aboagye
Last updated by author(s): Mar 21, 2022

# Reporting Summary

Nature Research wishes to improve the reproducibility of the work that we publish. This form provides structure for consistency and transparency in reporting. For further information on Nature Research policies, see our Editorial Policies and the Editorial Policy Checklist.

## Statistics

For all statistical analyses, confirm that the following items are present in the figure legend, table legend, main text, or Methods section.

| n/a | Confirmed | |
|---|---|---|
| ☐ | ☒ | The exact sample size ($n$) for each experimental group/condition, given as a discrete number and unit of measurement |
| ☐ | ☒ | A statement on whether measurements were taken from distinct samples or whether the same sample was measured repeatedly |
| ☐ | ☒ | The statistical test(s) used AND whether they are one- or two-sided<br>*Only common tests should be described solely by name; describe more complex techniques in the Methods section.* |
| ☒ | ☐ | A description of all covariates tested |
| ☐ | ☒ | A description of any assumptions or corrections, such as tests of normality and adjustment for multiple comparisons |
| ☐ | ☒ | A full description of the statistical parameters including central tendency (e.g. means) or other basic estimates (e.g. regression coefficient) AND variation (e.g. standard deviation) or associated estimates of uncertainty (e.g. confidence intervals) |
| ☒ | ☐ | For null hypothesis testing, the test statistic (e.g. $F$, $t$, $r$) with confidence intervals, effect sizes, degrees of freedom and $P$ value noted<br>*Give P values as exact values whenever suitable.* |
| ☒ | ☐ | For Bayesian analysis, information on the choice of priors and Markov chain Monte Carlo settings |
| ☒ | ☐ | For hierarchical and complex designs, identification of the appropriate level for tests and full reporting of outcomes |
| ☒ | ☐ | Estimates of effect sizes (e.g. Cohen's $d$, Pearson's $r$), indicating how they were calculated |

*Our web collection on statistics for biologists contains articles on many of the points above.*

## Software and code

Policy information about availability of computer code

| | |
|---|---|
| Data collection | No software was used for data collection. |
| Data analysis | MATLAB, FreeSurfer, R |

For manuscripts utilizing custom algorithms or software that are central to the research but not yet described in published literature, software must be made available to editors and reviewers. We strongly encourage code deposition in a community repository (e.g. GitHub). See the Nature Research guidelines for submitting code & software for further information.

## Data

Policy information about availability of data

All manuscripts must include a data availability statement. This statement should provide the following information, where applicable:

- Accession codes, unique identifiers, or web links for publicly available datasets
- A list of figures that have associated raw data
- A description of any restrictions on data availability

The radiomics data generated in this study have been deposited into the Mendeley database under the accession code DOI: 10.17632/rpztyz22df.1. All the other data supporting the findings of this study are available within the article and its supplementary information files and from the corresponding author upon reasonable request.

# Field-specific reporting

Please select the one below that is the best fit for your research. If you are not sure, read the appropriate sections before making your selection.

☒ Life sciences ☐ Behavioural & social sciences ☐ Ecological, evolutionary & environmental sciences

For a reference copy of the document with all sections, see nature.com/documents/nr-reporting-summary-flat.pdf

# Life sciences study design

All studies must disclose on these points even when the disclosure is negative.

**Sample size**

*Describe how sample size was determined, detailing any statistical methods used to predetermine sample size OR if no sample-size calculation was performed, describe how sample sizes were chosen and provide a rationale for why these sample sizes are sufficient.*

**Data exclusions**

From ADNI database, we included all subjects for whom baseline MRI data (T1w MP-RAGE sequence at 1.5 and 3T), age, and cognitive scores (Mini-Mental stage examination (MMSE) and the Logical Memory Delayed Recall Total (LDELTOTAL)), and cerebrospinal fluid based biomarkers (Aβ, tau and ptau) were available. Subjects without structural MRI and MMSE+LDELTOTAL+ Aβ, tau and ptau were excluded for further evaluation.
Similarly, from the OASIS database, we included subjects for whom baseline T1w sequence, age and MMSE score were available.
The IMC cohort includes subjects who underwent clinical amyloid PET imaging as part of their diagnostic workup. Of the 396 patients who had an Amyloid PET scan between December 2013 and June 2019, we included those who had an MRI scan available acquired between 3-6 months after the Amyloid PET scan and received a clinical neuropsychological assessment which included the administration of the Logical Memory Test and the MMSE administered within 12 months of MRI scanning.

**Replication**

The repeatability of the method was tested on a second T1w MRI scan obtained on the same day of the baseline scan used for training the model. Based on the reporting guidelines by Koo and Li, a one-way random effects, absolute agreement, single rater/measurement interclass correlation coefficient (ICC) was evaluated and was 0.83, 0.89, 0.83 and 0.82 for ApV1, ApV1s, ApV2 and ApV2s, respectively.

**Randomization**

The model was trained on 1.5T T1w MRI scans obtained from ADNI. After a stratified randomisation, 70% of data was used for training and 30% for validation.

**Blinding**

Subjects were randomly allocated for data analysis.

# Reporting for specific materials, systems and methods

We require information from authors about some types of materials, experimental systems and methods used in many studies. Here, indicate whether each material, system or method listed is relevant to your study. If you are not sure if a list item applies to your research, read the appropriate section before selecting a response.

## Materials & experimental systems

| n/a | Involved in the study | |
|---|---|---|
| ☒ | ☐ | Antibodies |
| ☒ | ☐ | Eukaryotic cell lines |
| ☒ | ☐ | Palaeontology and archaeology |
| ☒ | ☐ | Animals and other organisms |
| ☐ | ☒ | Human research participants |
| ☒ | ☐ | Clinical data |
| ☒ | ☐ | Dual use research of concern |

## Methods

| n/a | Involved in the study | |
|---|---|---|
| ☒ | ☐ | ChIP-seq |
| ☒ | ☐ | Flow cytometry |
| ☐ | ☒ | MRI-based neuroimaging |

## Human research participants

Policy information about studies involving human research participants

**Population characteristics**

Data used in this work were obtained from the Alzheimer's Disease Neuroimaging Initiative (ADNI) database (www.loni.ucla.edu/ADNI), launched in 2003 as a public-private partnership, led by Principal Investigator Michael W. Weiner, MD. The primary goal of ADNI is to test whether serial MRI, PET, other biological markers, and clinical and neuropsychological assessment can be combined to measure the progression of MCI and early AD. For up-to-date information, see www.adni-info.org. From this database, all subjects for whom baseline MRI data (T1w magnetization-prepared rapid acquisition with gradient echo (MP-RAGE) sequence at 1.5T), age, and cognitive scores (Mini-Mental stage examination (MMSE)25, a brief screening test for cognitive status and the Logical Memory Delayed Recall Total (LDELTOTAL)26, a measure of verbal episodic memory), CSF based biomarkers (Aβ, tau and ptau) were available have been included.
For the diagnostic classification at baseline, the method was trained on:
- 783 subjects scanned at 1.5T (ADNI1 cohort). They were grouped as 216 healthy controls, 208 subjects with MCI due to AD (referred to as MCIAD in the text), 181 AD, 94 patients with Frontotemporal Dementia (FTD), and 84 with Parkinson's disease (PD). In particular, based on the data obtained from the ADNI database, two new groups of subjects were defined:

a) the nADrp group, which contains subjects who do not show any pathology related to AD (healthy controls, PD and FTD were included here) and
b) the ADrp group which, on the contrary, contains subjects with MCI due to AD and AD patients.
The method was externally tested on:
1) An unseen 1.5T dataset obtained from the Open Access Series of Imaging Studied (OASIS) consortium (https://www.oasis-brains.org/) of 64 subjects for whom baseline T1w sequence, age and MMSE score were available (53 CN and 11 AD).
2) An unseen 3T dataset of 402 subjects obtained from the ADNI3 cohort for whom baseline T1w sequence, age, cognitive scores and CSF related biomarkers were available (172 CN, 161 MCIAD and 69 AD).
3) The IMC cohort: 83 patients with Atypical Presentations who underwent clinical amyloid PET imaging at the Imperial Memory Centre (IMC, London, UK) as part of their diagnostic workup with a 1.5T T1w MRI scan. Of the 396 patients who had an Amyloid PET scan between December 2013 and June 2019, those (n=83) who had an MRI scan available acquired between 3-6 months after the Amyloid PET scan and received a clinical neuropsychological assessment which included the administration of the Logical Memory Test, were included to the study. Of these, a subgroup of 22 patients also had an MMSE administered within 12 months of MRI scanning. At the Memory Centre, the decision to perform clinical Amyloid-PET scan is made by consensus within the Cognitive Neuroradiology Multidisciplinary Team27 and referral to Amyloid imaging is in line with the Appropriate Use Criteria published by the Amyloid Imaging Taskforce28. These criteria recommend the use of clinical amyloid PET in three main categories of patients: (1) with persistent/progressive unexplained MCI; (2) with atypical course or aetiologically mixed presentation; (3) with early age of onset. Moreover, patients undergoing clinical API should report objective cognitive impairment with substantial diagnostic uncertainty following a comprehensive evaluation28. For the IMC cohort, mainly employed for the classification/evaluation of earlier disease using structural MRI, all images were visually read as 'amyloid-positive' (AMY+, N = 45) or 'amyloid-negative' (AMY-, N = 38) by an experienced nuclear medicine radiologist using greyscale images. All AMY+ patients received a clinical diagnosis of AD. AMY- patients were either diagnosed with another neurodegenerative disease (progressive non-AD MCI (n=4), MCI due to hypertensive microvascular disease (n=1), unspecified neurodegenerative disease (NDG) (n=1), MCI due to previous stroke (n=1), NDG with Parkinsonian features (n=1), Lewy body dementia (n=1), tauopathy (n=1), normal pressure hydrocephalus (n=1), isolated cerebral amyloid angiopathy (n=1)) or with a non-neurodegenerative condition (e.g., depression).

Recruitment

ADNI and OASIS cohorts belong to online available databases. At the Memory Clinic, the decision to perform clinical Amyloid-PET scan is made by consensus within the Cognitive Neuroradiology Multidisciplinary Team25 (Kolanko et al., 2020) and referral to Amyloid imaging is in line with the Appropriate Use Criteria published by the Amyloid Imaging Taskforce26. These criteria recommend the use of clinical amyloid PET in three main categories of patients: (1) with persistent/progressive unexplained MCI; (2) with atypical course or aetiologically mixed presentation; (3) with early age of onset. Moreover, patients undergoing clinical API should report objective cognitive impairment with substantial diagnostic uncertainty following a comprehensive evaluation26. For the IMC cohort, all images were visually read as 'amyloid-positive' (AMY+) or 'amyloid-negative' (AMY-) by an experienced nuclear medicine radiologist using greyscale images. All AMY+ patients received a clinical diagnosis of AD, AMY- patients, instead, were either diagnosed with another neurodegenerative disease (progressive non-AD MCI (n=4), MCI due to hypertensive microvascular disease (n=1), unspecified neurodegenerative disease (NDG) (n=1), MCI due to previous stroke (n=1), NDG with Parkinsonian features (n=1), Lewy body dementia (n=1), tauopathy (n=1), normal pressure hydrocephalus (n=1), isolated cerebral amyloid angiopathy (n=1)) or with a non-neurodegenerative condition (e.g., depression).

Ethics oversight

For the IMC cohort, we received ethical approval by the Camden and Kings Cross UK Research Ethics Committee (IRAS n. 273966) to perform retrospective anonymised and unlinked analysis of all clinical data (including MR images), provided that these were anonymised at source by a member of the clinical care team.
In particular, the study protocol states that: "For all patients undergoing Amyloid PET at Imperial College Healthcare NHS Trust (ICHT) from December 2013 to January 2023 we will perform retrospective anonymised and unlinked analysis of clinically collected data. This will be anonymised at source by members of the clinical care team. The data will be unlinked and there will be no prospective element to this data collection."

The other two cohorts of subjects (ADNI and OASIS) belong two publicly available datasets. All participants provided informed consent. Details about the Ethics statement of the ADNI study population can be found at: https://adni.loni.usc.edu". Details about the Ethics statement of the OSASIS study population can be found at: https://www.oasis-brains.org/#data.

Note that full information on the approval of the study protocol must also be provided in the manuscript.

# Magnetic resonance imaging

## Experimental design

Design type: n/a

Design specifications: n/a

Behavioral performance measures: n/a

## Acquisition

| | |
|---|---|
| Imaging type(s) | structural |
| Field strength | 1.5 T |
| Sequence & imaging parameters | gradient recalled echo |
| Area of acquisition | whole brain |

Diffusion MRI ☐ Used ☒ Not used

## Preprocessing

| | |
|---|---|
| Preprocessing software | MATLAB 2019b |
| Normalization | n/a |
| Normalization template | n/a |
| Noise and artifact removal | n/a |
| Volume censoring | n/a |

## Statistical modeling & inference

| | |
|---|---|
| Model type and settings | n/a |
| Effect(s) tested | n/a |

Specify type of analysis: ☐ Whole brain ☒ ROI-based ☐ Both

Anatomical location(s): With FreeSurfer, we performed the segmentation of the brain in 115 regions (recon-all function)

| | |
|---|---|
| Statistic type for inference (See Eklund et al. 2016) | n/a |
| Correction | n/a |

## Models & analysis

| n/a | Involved in the study |
|---|---|
| ☒ | ☐ Functional and/or effective connectivity |
| ☒ | ☐ Graph analysis |
| ☒ | ☐ Multivariate modeling or predictive analysis |
